# Duration of Quarantine after Scarlet Fever

**Published:** 1913-11

**Authors:** 


Duration of Quarantine After Scarlet Fever. Lewis A. Sexton, New York *Arch. of Ped.,* May, 1913, speaks from an experience of 10,093 cases in six years. While the Sanitary Code requires a quarantine of thirty-five days, at the Willard Parker Hospital, forty-two days were required on the average and no case was discharged while a rhinorrhœa persisted. Notwithstanding these precautions, sixteen return cases were noted, apparently due to infection from discharged cases, all of which had subsequently developed a nasal discharge. In only two was desquamation noted after discharge and this was slight. The author denies the old notion that the desquamation is infectious, and lays stress on the danger of nasal and aural discharges.

---

R. S. Titus, Boston, *Boston M. and S. Jour.,* September 11, 1913, reports recovery after removal of Fibroma of Ovary, weighing 35 pounds.

